# Risk and prognosis for brain metastasis in primary metastatic cervical cancer patients: A population-based study

**DOI:** 10.1515/med-2025-1165

**Published:** 2025-08-22

**Authors:** Jiao Wu, Qing Xu, Huixia Huang, Yangyang Pang, Haoran Li, Xi Cheng

**Affiliations:** Department of Gynecological Oncology, Fudan University Shanghai Cancer Center, Shanghai, 200032, China; Department of Oncology, Shanghai Medical College, Fudan University, Shanghai, 200032, China; Department of Urology, Shanghai Xuhui Central Hospital, Shanghai, China; Department of Gynecological Oncology, Fudan University Shanghai Cancer Center, 270 Dong’an Road, Shanghai, 200032, China; Department of Oncology, Shanghai Medical College, Fudan University, 270 Dong’an Road, Shanghai, 200032, China

**Keywords:** primary metastatic cervical cancer, brain metastasis, risk factors, overall survival

## Abstract

**Purpose:**

The purpose of this study was to evaluate the risk and prognostic factors of stage IVB cervical cancer with brain metastasis from a population-based database, the Surveillance, Epidemiology and End Results (SEER).

**Patients and methods:**

Cervical cancer patients initially diagnosed with brain metastasis between 2010 and 2019 were included in this study. The risk factors of developing brain metastasis were evaluated by logistic regression model with corresponding 95% confidence interval (95% CI). Survival analysis was performed through the Kaplan–Meier method, log-rank test, and Cox proportional hazards model.

**Results:**

A total of 88 (88/25,476, 0.35%) cervical cancer patients initially diagnosed with brain metastasis between 2010 and 2019 were retrieved. Accompanied with lung, bone, or liver metastasis (all *P* < 0.001) was shown to be independent risk factors for developing brain metastasis. Patients with brain metastasis indicated a poor prognosis (*P* < 0.001, hazards ratio [HR] = 2.84, 95% CI = 1.71–4.72) with a 2.84-fold elevated risk of death compared with patients without brain metastasis. The median survival month for patients with brain metastasis was 6 months, which is much shorter compared with the lung (9 months) or liver (8.5 months) or bone (11 months) metastasis group. Along with lower tumor grade (*P* = 0.001, HR = 0.27, 95% CI = 0.09–0.76) and with bone metastasis (*P* = 0.007, HR = 2.74, 95% CI = 1.33–5.67) demonstrated poor overall survival outcomes in patients with brain metastasis, with a 3.7- and 1.33-fold higher risk of death, respectively. In terms of treatment modality, chemoradiotherapy tended to prolong the survival of stage IVB cervical cancer patients with brain metastasis (*P* = 0.001, HR = 0.17, 95% CI = 0.06–0.48), with an 83% reduction in the risk of death.

**Conclusion:**

In conclusion, the prognosis of stage IVB cervical cancer patients with brain metastasis remains poor. Chemoradiotherapy may provide survival benefits, which deserves large-scale prospective clinical trials to confirm.

## 1 Introduction

Cervical cancer ranked as fourth most prevalent and fourth leading lethal malignancy in women worldwide, with nearly 604,000 new cases and 342,000 deaths in 2020 [1,2]. According to the National Comprehensive Cancer Network Guidelines, the primary treatment of early-stage cervical cancer is either surgery or radiotherapy. About 80% of women with early-stage disease (stage I–II) and 60% of women with stage III disease can be cured with effective treatment (including surgery and concurrent chemoradiotherapy) [3]. Nevertheless, patients who develop distant metastases (stage IVB) are rarely curable, with 5-year survival rate less than 20%. Even worse, approximately 50% of these patients show a fatal outcome within 1 year [4,5].

In light of previous studies, stage IVB cervical cancer was divided into two metastatic types. Lymphatic metastasis is defined as all the involved sites being lymph nodes outside of the pelvic, including para-aortic lymph nodes, while the other metastasis as hematogenous metastasis. As reported, hematogenous metastasis presented with a 5.3-fold higher risk of death than lymphatic metastasis [6]. For stage IVB cervical cancer patients, lung, liver, and bone are the most frequently involved sites of hematogenous metastasis. Despite brain metastasis constituting the most common type of intracranial tumor, those from cervical cancer is a rare event, with an incidence rate ranging from 0.4 to 2.3%. Brain metastasis is usually found later in the course of the disease with a median survival time of 1–8 months, often indicating an unfavorable prognosis [7–9]. There are currently no satisfactory treatment guidelines being established. The main management for brain metastasis is composed of surgery, local radiotherapy (whole brain radiation therapy [WBRT] and stereotactic radiosurgery [SRS]), systemic chemotherapy, or simply symptom control, similar to those for the other visceral metastasis [8,10].

Up to now, cervical cancer complicated with progressive distant metastasis indicates poor prognosis and causes increasing cancer-related mortality. Therefore, management with these patients remains a rather intractable challenge for physicians. As a small branch of Stage IVB cervical cancer, patients with brain metastasis were not systematically studied due to the paucity of clinical data and low level of the follow-up quality. Either retrospective or prospective clinical study concentrating on the analysis of risk factors and optimizing treatment plan is urgently desired for the sake of clinical application. In the present study, we retrieved information of cervical cancer patients with brain metastasis from the Surveillance, Epidemiology, and End Results (SEER) database. We analyzed the risk factors for developing brain metastasis and prognostic factors for overall survival (OS) of stage IVB cervical cancer with brain metastasis, which may contribute to clinical practice.

## 2 Materials and methods

### 2.1 Data source

The SEER database, a population-based registry, is sponsored by the National Cancer Institute. With 18 population-based cancer registries, the SEER program covers approximately 28% of the cancer registries from the United States [11]. The National Cancer Institute’s SEER*Stat software (version 8.3.5; Surveillance Research Program, National Cancer Institute SEER*Stat software, https://seer.cancer.gov/) was used to extract data after access permitted by signing an agreement. In view of that SEER database is an open public database, written informed consent cannot be assessed.

### 2.2 Study population

The retrospective clinical data of stage IVB cervical cancer patients from 2010 to 2019 was retrieved from the recent SEER-18 database. We limited this study to patients diagnosed between 2010 and 2019 as detailed information about site-specific metastasis was not recorded before 2010. Since 2010, the SEER data provide only four specific sites of metastases (bone, brain, liver, and lung). Other sites of metastasis are not documented currently. We included site codes C53.0-C53.1, C53.8, and C53.9 to identify primary cervical cancer based on the International Classification of Diseases for oncology, third Edition. The following baseline demographic and clinicopathologic characteristics of patients were collected: age at diagnosis; year of diagnosis, between 2010 and 2019; race, White, Black, or others including Asian or Pacific Islander; American Indian/Alaska Native; marital status, married or unmarried; tumor grade, I–II (including G1 or well-differentiated, G2 or moderately-differentiated), III–IV (including G3 or poorly-differentiated, G4 or undifferentiated or anaplastic); tumor histology including squamous cell carcinoma, adenocarcinoma, and other types including epithelial neoplasms, transitional cell papillomas and carcinomas, cystic, mucinous and serous neoplasms, complex epithelial neoplasms, complex mixed and stromal neoplasms, and unspecified neoplasms; American Joint Committee on Cancer (AJCC) stage; tumor size (the SEER database records the most accurate measurement of a solid primary tumor, usually measured on the surgical resection specimen); site-specific metastasis, including lung, bone, liver, and brain; cause-specific death classification; vital status; and survival months. Patients with visceral metastasis means patients were diagnosed with any site-specific metastasis including lung, bone, liver, and brain.

In addition, treatment data retrieved for each case included chemotherapy, radiotherapy, and surgery (including primary sites and metastatic sites). Patients were classified into two groups in our final analysis including with brain metastasis group and without brain metastasis group.

### 2.3 Statistical analysis

Data analysis was executed using R (version 4.2.1). The risk factors of developing brain metastasis were evaluated by logistic regression model with corresponding 95% confidence interval (95% CI). The primary outcome of the survival analysis was the OS (survival months), which was defined from the time of diagnosis of uterine cervical cancer to causes of cancer-specific death. Estimate of OS was performed using the Kaplan–Meier method and the log-rank test. The survival curves were made by Graph Pad Prism. Cox regression models were applied to estimate the impact of clinical factors for patients’ survival. A probability value of less than 0.05 was considered statistically significantly different.

## 3 Results

### 3.1 Risk analysis

**Figure 1:**
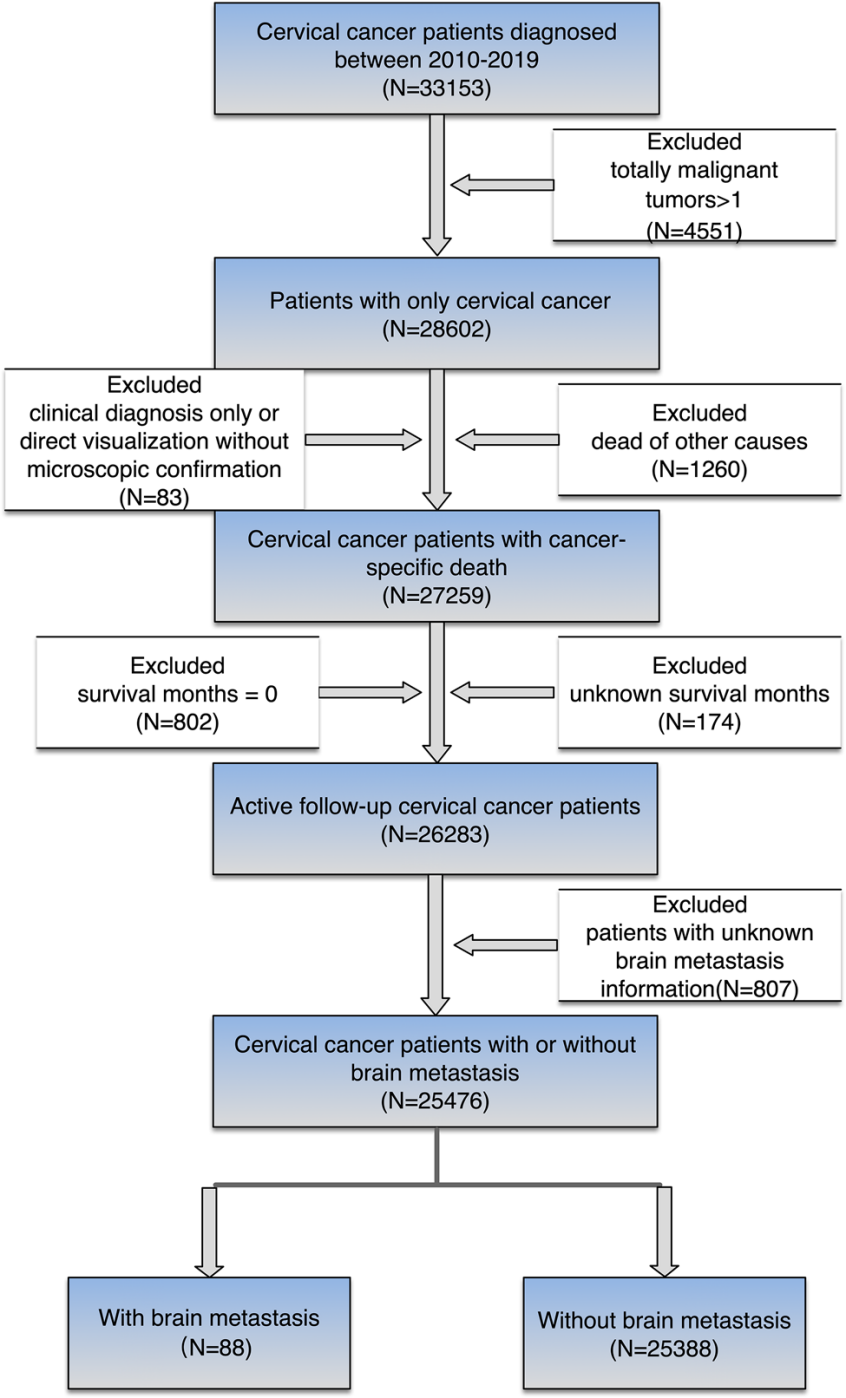
Flowchart of this study.

The screening flow-chart is shown in Figure 1. Between 2010 and 2019, a total of 33,153 patients with cervical cancer were identified from the SEER database. Patients with more than one primary malignant tumor (*N* = 4,551) and clinical diagnosis only or direct visualization without microscopic confirmation (*N* = 83) were excluded from this study. In addition, patients who died of other causes (*N* = 1,260), with survival months of 0 (*N* = 802) or unknown survival months (*N* = 174), and unknown brain metastasis information (*N* = 807) were also filtered out. Following the screening procedure, a total of 25,476 cervical cancer patients were finally included in this study, among whom 88 patients (88/25,476, 0.35%) were diagnosed with brain metastasis, while 25,388 patients were diagnosed without brain metastasis (Table 1). To find out the independent risk factors in developing brain metastasis in patients with cervical cancer, the multivariate logistic regression analysis was performed. Patients with lung metastasis (without vs with, *P* < 0.001, odds ratio [OR] = 1.037, 95% CI = 1.033–1.041), liver metastasis (without vs with, *P* < 0.001, OR = 1.134, 95% CI = 1.109–1.161), or bone metastasis (without vs with, *P* < 0.001, OR = 1.025, 95% CI = 1.020–1.030) had a 1.037-, 1.134-, and 1.025-fold increased risk of brain metastases, respectively (Table 1). Nevertheless, age older than 65 (≤39 vs ≥65, *P* = 0.001, OR = 0.996, 95% CI = 0.994–0.999) were associated with decreased risk of developing brain metastasis, with a 0.4% risk reduction.

**Table 1:** Baseline demographic and clinicopathologic characteristics and risk analysis in cervical cancer patients developing brain metastasis

Characteristic (%)	Without brain metastasis (*n* = 25,388)	With brain metastasis (*n* = 88)	Multivariate analysis	
**Age at diagnosis**				
≤39	11,005 (43.3)	32 (36.4)	1	
>40, ≤64	10,479 (41.3)	44 (50.0)	0.999 (0.998–1.001)	0.3
≥65	3,904 (15.4)	12 (13.6)	0.996 (0.994–0.999)	**0.001**
**Race**				
White	18,876 (74.4)	69 (78.4)	1	
Black	3,197 (12.6)	13 (14.8)	0.999 (0.997–1.001)	0.468
Others*	2,987 (11.8)	5 (5.7)	0.998 (0.996–1.001)	0.133
Unknown	328 (1.3)	1 (1.1)		
**Marital status**				
Unmarried	12,839 (50.6)	45 (51.1)	1	
Married	11,023 (43.4)	36 (40.9)	1.000 (0.999–1.002)	0.723
Unknown	1,526 (6.0)	7 (8.0)		
**Grade**				
I–II	8,736 (34.4)	12 (13.6)	1	
III–IV	5,996 (23.6)	36 (40.9)	1.002 (0.999–1.004)	0.082
Unknown	10,656 (42.0)	40 (45.5)		
**Histology**				
SCC	16,486 (64.9)	53 (60.2)	1	
Adenocarcinoma	6,105 (24.0)	17 (19.3)	1.000 (0.998–1.002)	0.825
Other*	2,797 (11.0)	18 (20.5)	1.001 (0.998–1.003)	0.509
**Tumor size (cm)**				
≤2	10,486 (41.3)	22 (25.0)	1	
>2, ≤4	42 (0.2)	0 (0.0)	0.999 (0.982–1.017)	0.916
>4	66 (0.3)	1 (1.1)	1.014 (1.000–1.028)	0.054
Unknown	14,794 (58.3)	65 (73.9)		
**AJCC T**				
T1	8,149 (32.1)	5 (5.7)	1	
T2	3,135 (12.3)	13 (14.8)	1.002 (1.000–1.004)	0.11
T3	2,225 (8.8)	15 (17.0)	1.000 (0.997–1.003)	0.795
T4	523 (2.1)	5 (5.7)	1.000 (0.995–1.006)	0.866
Unknown	11,356 (44.7)	50 (56.8)		
**AJCC N**				
N0	10,316 (40.6)	12 (13.6)	1	
N1	3,697 (14.6)	31 (35.2)	1.002 (1.000–1.004)	0.106
Unknown	11,375 (44.8)	45 (51.1)		
**Lung metastasis**				
No	24,380 (96.0)	38 (43.2)	1	
Yes	957 (3.8)	46 (52.3)	1.037 (1.033–1.041)	**<0.001**
Unknown	51 (0.2)	4 (4.5)		
**Liver metastasis**				
No	24,874 (98.0)	69 (78.4)	1	
Yes	493 (1.9)	15 (17.0)	1.134 (1.109–1.161)	**<0.001**
Unknown	21 (0.1)	4 (4.5)		
**Bone metastasis**				
No	24,802 (97.7)	60 (68.2)	1	
Yes	572 (2.3)	27 (30.7)	1.025 (1.020–1.030)	**<0.001**
Unknown	14 (0.1)	1 (1.1)		

Abbreviations: SCC, squamous cell carcinoma; OR, odds ratio; CI, confidence interval; AJCC, American Joint Committee on Cancer. The results were adjusted for age at diagnosis, race, grade, marital status, histology, AJCC T, AJCC N, lung or liver or bone metastasis, and the significant results in bold, if *P* < 0.05. *Race – others (Asian or Pacific Islander; American Indian/Alaska Native); ^†^histology – others (epithelial neoplasms, NOS; transitional cell papillomas and carcinomas; cystic, mucinous and serous neoplasms; complex epithelial neoplasms; complex mixed and stromal neoplasms; unspecified neoplasms). The results are in bold if *P* < 0.05.

### 3.2 Survival analysis

**Figure 2:**
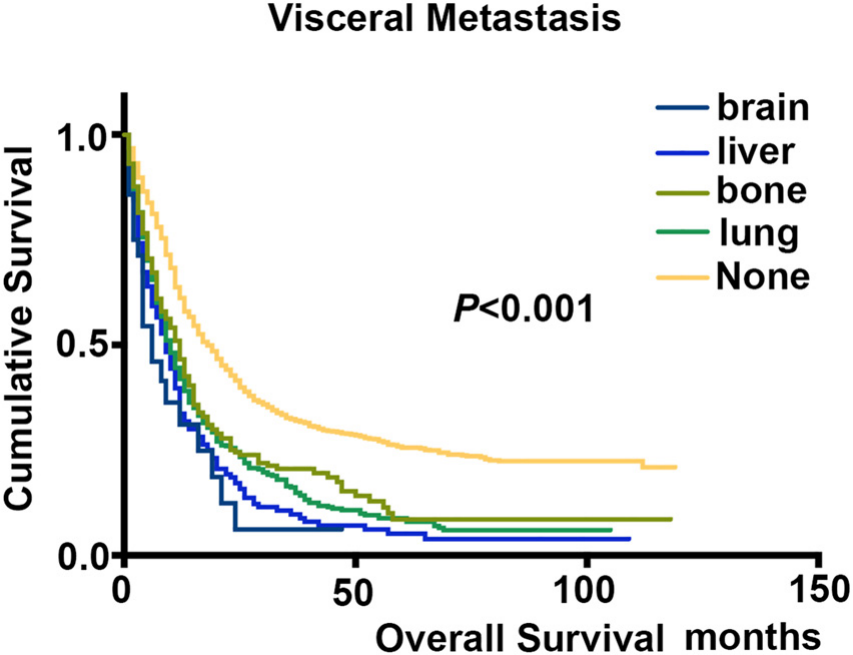
Kaplan–Meier analyses with the log-rank for the impact of different visceral metastasis on OS in stage IVB cervical cancer patients.

In order to analyze the impact of different visceral metastasis sites on survival, we focused on patients with stage IVB cervical cancer. As expected, patients with brain metastasis indicated a poor prognosis with a median survival month of 6, which is much shorter compared with the lung (9 months) or liver (8.5 months) or bone (11 months) metastasis group. In consistent with above results, we observed an inferior survival outcome of patients with brain metastasis (*P* < 0.001, hazards ratio [HR] = 2.84, 95% CI = 1.71–4.72) in multivariate analysis, with a 2.84-fold elevated risk of death compared with patients without brain metastasis (Table 2, Figure 2).

**Table 2:** Survival analysis of stage IVB cervical cancer patients with visceral metastasis

Characteristic	Survival months (median)	Univariate analysis		Multivariate analysis	
**Visceral metastasis**		HR (95% CI)	*P*-value	HR (95%CI)	*P*-value
None	17	1		1	
Lung	9	1.85 (1.61–2.12)	**<0.001**	1.38 (1.20–1.59)	**<0.001**
Liver	8.5	2.13 (1.73–2.62)	**<0.001**	1.79 (1.44–2.22)	**<0.001**
Bone	11	1.75 (1.44–2.12)	**<0.001**	1.67 (1.37–2.04)	**<0.001**
Brain	6	3.18 (1.93–5.22)	**<0.001**	2.84 (1.71–4.72)	**<0.001**
More than one site	5	2.91 (2.55–3.32)	**<0.001**	2.36 (2.06–2.71)	**<0.001**

The multivariate results were adjusted for age at diagnosis, race, marital status, grade, histology, AJCC T, AJCC N, surgery, chemoradiotherapy and the significant results are in bold, if *P* < 0.05.

**Figure 3:**
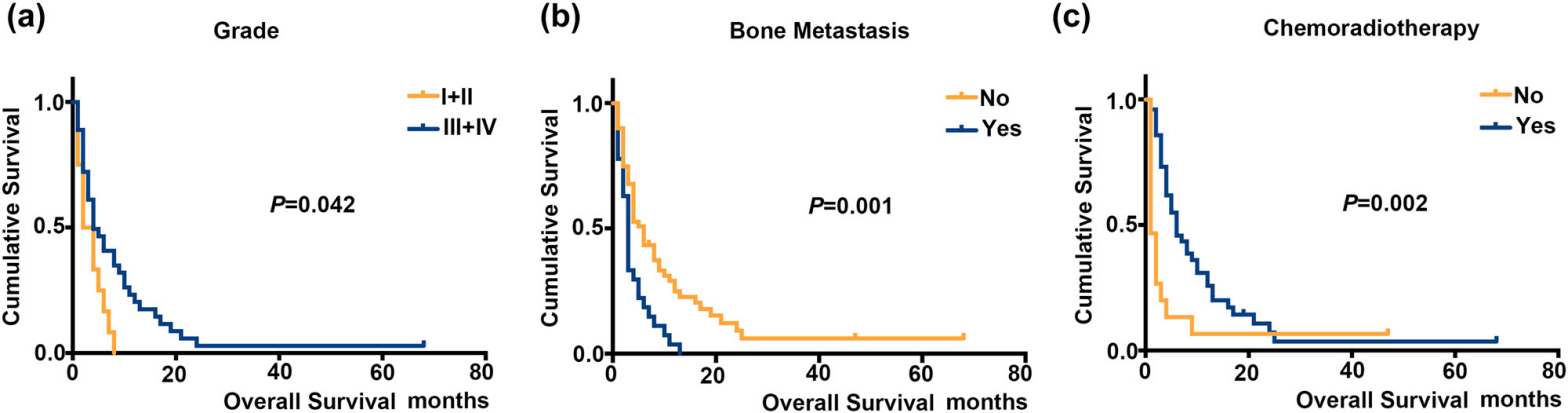
Kaplan–Meier analyses with the log-rank for OS in stage IVB cervical cancer patients with brain metastasis: (a) grade I–II vs III–IV, (b) with vs without bone metastasis, and (c) with vs without chemotherapy.

Furthermore, to clarify the association of demographic and clinicopathologic characteristics with cancer-specific OS in stage IVB cervical cancer patients with brain metastasis, univariate and multivariate analysis were performed for adjusting variants including age at diagnosis, race, marital status, grade, histology, tumor size, AJCC stage, visceral metastasis, surgery, radiotherapy, and chemotherapy (Table 3). In univariate analysis, we found several factors significantly associated with OS. Tumor grade (I–II vs III–IV, *P* = 0.042, HR = 0.49, 95% CI = 0.25–0.97) was associated with a 51% reduction in the risk of death. Conversely, the presence of bone metastasis (with vs without, *P* = 0.001, HR = 2.30, 95% CI = 1.41–3.77) was linked to a 2.30-fold increase in the risk of death. Additionally, patients receiving chemoradiotherapy (with vs without, *P* = 0.018, HR = 0.37, 95% CI = 0.20–0.68) had a 63% lower risk of death compared to those not receiving this treatment (Table 3, Figure 3). In multivariate analysis, patients with lower tumor grade (*P* = 0.001, HR = 0.27, 95% CI = 0.09–0.76), AJCC T3 stage (*P* = 0.005, HR = 11.17, 95% CI = 2.10–59.45), with bone metastasis (*P* = 0.007, HR = 2.74, 95% CI = 1.33–5.67) demonstrated worse OS outcomes, with a 3.7-, 11.17- and 2.74-fold higher risk of death, respectively. Moreover, chemoradiotherapy tended to prolong the survival of stage IVB cervical cancer patients with brain metastasis (*P* = 0.001, HR = 0.17, 95% CI = 0.06–0.48), with an 83% reduction in the risk of death.

**Table 3:** Association of demographic and clinicopathologic characteristics with cancer-specific OS in cervical cancer patients with brain metastasis

Characteristic	Univariate analysis		Multivariate analysis	
	HR (95% CI)	*P*-value	HR (95% CI)	*P*-value
**Age at diagnosis**				
≤39	1		1	
>40, ≤64	0.91 (0.55–1.50)	0.708	1.46 (0.78–2.76)	0.241
≥65	0.99 (0.48–2.06)	0.979	1.04 (0.40–2.70)	0.939
**Race**				
White	1		1	
Black	0.92 (0.47–1.80)	0.801	0.66 (0.25–1.76)	0.406
Others*	0.85 (0.34–2.12)	0.727	1.18 (0.36–3.89)	0.783
**Marital status**				
Unmarried			1	
Married	0.94 (0.59–1.51)	0.802	1.50 (0.75–3.00)	0.25
**Grade**				
I–II	1		1	
III–IV	0.49 (0.25–0.97)	**0.042**	0.27 (0.09–0.76)	**0.013**
**Histology**				
SCC	1		1	
Adenocarcinoma	0.71 (0.39–1.32)	0.285	0.56 (0.21–1.49)	0.25
Other^†^	1.10 (0.61–1.97)	0.755	1.36 (0.60–3.11)	0.463
**Tumor size (cm)**				
≤2	1		1	
>2, ≤4	NA	NA	NA	NA
>4	0.99 (0.13–7.39)	0.995	0.50 (0.03–7.92)	0.624
**AJCC T**				
T1	1		1	
T2	1.92 (0.68–5.42)	0.218	4.54 (0.86–24.03)	0.076
T3	1.33 (0.48–3.67)	0.584	11.17 (2.10–59.45)	**0.005**
T4	2.03 (0.58–7.14)	0.27	1.85 (0.36–9.33)	0.459
**AJCC N**				
N0	1			
N1	1.20 (0.60–2.40)	0.6	1.65 (0.68–4.00)	0.595
**Lung metastasis**				
No	1		1	
Yes	1.11 (0.69–1.77)	0.673	0.95 (0.46–1.97)	0.9
**Liver metastasis**				
No	1		1	
Yes	1.22 (0.68–2.21)	0.507	1.25 (0.52–3.04)	0.617
**Bone metastasis**				
No	1		1	
Yes	2.30 (1.41–3.77)	**0.001**	2.74 (1.33–5.67)	**0.006**
**Surgery**				
No	1		1	
Yes^‡^	0.64 (0.28–1.48)	0.301	0.87 (0.29–2.60)	0.808
**Chemoradiotherapy**				
No	1		1	
Chemotherapy	0.56 (0.25–1.25)	0.157	0.70 (0.20–2.49)	0.58
Radiotherapy	0.76 (0.34–1.69)	0.501	0.48 (0.17–1.43)	0.187
Chemoradiotherapy	0.37 (0.20–0.68)	**0.002**	0.17 (0.06–0.48)	**0.001**

Abbreviations: OS, overall survival; SCC, squamous cell carcinoma; HR, hazards ratio; CI, confidence interval; AJCC, American Joint Committee on Cancer. The multivariate results were adjusted for age at diagnosis, race, marital status, grade, histology, AJCC T, AJCC N, lung or liver or bone metastasis, surgery, radiotherapy, chemotherapy and the significant results in bold, if *P* < 0.05. *Race – others (Asian or Pacific Islander; American Indian/Alaska Native) ^†^Histology – others (epithelial neoplasms, NOS; transitional cell papillomas and carcinomas; cystic, mucinous and serous neoplasms; complex epithelial neoplasms; complex mixed and stromal neoplasms; unspecified neoplasms). ^‡^Surgery – Yes (surgery of primary sites).

### 3.3 Stratified analysis

In clinical practice, multiple factors may influence the treatment decision. To select the optimal patients with brain metastasis amenable to chemoradiotherapy, the stratified survival analysis was also conducted according to sub-classification of stage IVB cervical cancer patients with brain metastasis. We performed the multivariate Cox regression analysis of chemoradiotherapy with adjusted clinical variables including age at diagnosis, race, marital status, grade, histology, AJCC T stage, AJCC N stage, visceral metastasis as shown in Table 4. In sum, chemoradiotherapy was recommended for white people (*P* < 0.001, HR = 0.04, 95% CI = 0.02–0.08) with a 96% reduction in the risk of death, significantly reducing the risk of death by 98% for squamous cell carcinoma (*P* < 0.001, HR = 0.02, 95% CI = 0.00–0.13) and by 99% for adenocarcinoma (*P* < 0.001, HR = 0.01, 95% CI = 9.39 × 10^−4^–0.04). Additionally, for patients with lymphatic metastasis (*P* = 0.001, HR = 2.36 × 10^−3^, 95% CI = 9.87 × 10^−4^–0.01), the treatment reduces the mortality risk by approximately 99.764%. In cases of lung metastasis (*P* < 0.001, HR = 0.02, 95% CI = 0.002–0.14), the death risk is also reduced by 98%. For patients without liver metastasis (*P* = 0.005, HR = 0.31, 95% CI = 0.14–0.70), the reduction in mortality risk is 69%. These benefits have been observed across all age groups, marital statuses, and event states of bone metastasis.

**Table 4:** Stratified analysis for associations between chemoradiotherapy and OS of stage IVB cervical cancer patients with brain metastasis

	Treatment			
Select covariates	Total/death	HR	95% CI	*P*
**Age at diagnosis**				
≤39	32/27	1.99 × 10^−3^	1.87 × 10^−5^–0.21	**0.009**
>40, ≤64	44/39	0.03	0.02–0.06	**<0.001**
≥65	12/10	NA	NA	NA
**Race**				
White	69/60	0.04	0.02–0.08	**<0.001**
Black	10/13	NA	NA	NA
Others*	5/0	NA	NA	NA
**Marital status**				
Unmarried	45/40	0.04	0.01–0.23	**<0.001**
Married	36/30	0.02	1.21 × 10^−3^–0.39	**0.009**
**Grade**				
I–II	12/12	NA	NA	NA
III–IV	36/34	6.67 × 10^−6^	4.11 × 10^−8^–1.08 × 10^−3^	**<0.001**
**Histology**				
SCC	53/48	0.02	0.00–0.13	**<0.001**
Adenocarcinoma	17/13	0.01	9.39 × 10^−4^–0.04	**<0.001**
Other^†^	18/15	1	0.29–3.45	1
**Tumor size (cm)**				
≤2	22/22	1	0.27–3.74	1
>2, ≤4	1/1	NA	NA	NA
>4	65/53	NA	NA	NA
**AJCC T**				
T1	5/5	NA	NA	NA
T2	13/13	NA	NA	NA
T3	15/15	1	0.06–15.99	1
T4	5/5	NA	NA	NA
**AJCC N**				
N0	12/1	NA	NA	NA
N1	31/31	2.36 × 10^−3^	9.87 × 10^−4^–0.01	**<0.001**
**Lung metastasis**				
No	38/31	1.02	0.07–14.00	0.99
Yes	46/41	0.02	0.002–0.14	**<0.001**
**Liver metastasis**				
No	69/58	0.31	0.14–0.70	**0.005**
Yes	15/14	1	0.00-INF	1
**Bone metastasis**				
No	60/48	0.12	0.03–0.50	**0.003**
Yes	27/27	1.16 × 10^−63^	3.45 × 10^−64^–3.88 × 10^−63^	**<0.001**

Abbreviations: OS, overall survival; SCC, squamous cell carcinoma; HR, hazards ratio; CI, confidence interval; AJCC, American Joint Committee on Cancer. The results were adjusted for age at diagnosis, race, marital status, grade, histology, AJCC T, AJCC N, lung or liver or bone metastasis, surgery, radiotherapy, chemotherapy and the significant results in bold, if *P* < 0.05. *Race – others (Asian or Pacific Islander; American Indian/Alaska Native). ^†^Histology – others (epithelial neoplasms, NOS; transitional cell papillomas and carcinomas; cystic, mucinous and serous neoplasms; complex epithelial neoplasms; complex mixed and stromal neoplasms; unspecified neoplasms).

## 4 Discussion

In this study, between 2010 and 2019, 88 stage IVB cervical cancer patients initially diagnosed with brain metastasis from the SEER database were available for analysis. The case numbers were much more substantial than in previous studies, which were mostly case reports [12,13]. This resulted in improved credibility of our research. Subsequently, we retrospectively evaluated the risk factors for developing brain metastasis and prognostic factors for OS in stage IVB cervical cancer patients with brain metastasis. To our knowledge, this study covered risk, survival, and treatment analysis for the first time and found that survival advantage significantly favors patients who underwent chemoradiotherapy in stage IVB cervical cancer patients with brain metastasis. These results were of important reference value for clinical diagnosis and treatment. In this study, the incidence rate of initially diagnosed brain metastasis was 0.35% (88/25,476) between 2010 and 2019, which was much less than recurrence with brain metastasis (1.2%). The result indicated possible different clinical characters between the two types [14,15]. Logically, brain metastasis deserves more detailed sub-classification and treatment modality optimization in clinical practice. Previous studies mainly focused on recurrence manifested with brain metastasis [13,16], while this study concentrated on initially diagnosed brain metastasis. Thereby, our research is of great novelty.

Further analysis revealed that patients with lung metastasis, liver metastasis, or bone metastasis were more likely to develop brain metastasis. The reason for this phenomenon may be that once cervical cancer patients developed liver, lung, or bone metastasis, cancer cells had already appeared in the blood, making it easier to penetrate the blood–brain barrier [10,17]. Surprisingly, in this study, age older than 65 were associated with decreased risk of developing brain metastasis. It seemed reasonable because previous study reported a high rate of cervical adenocarcinoma in younger patients with an increasing risk of lymph node metastasis, which resulted in a reduced survival rate compared with older women [18]. Brain metastasis can occur in any pathological type [19–21]. Pervious study observed an increased probability for developing brain metastasis in small cell carcinoma (SCC) of cervical cancer, which may be associated with a poor prognosis. Kim et al. reported a different outcome that SCC achieved more survival than other histology, which could be explained by the fact that cervical cancer patients with SCC were more likely to receive multimodal therapy [9]. However, in this study we found no statistical relationship between histology types and the occurrence and prognosis in stage IVB cervical cancer patients with brain metastasis.

Previous researchers reported that patients without brain metastasis are associated with a better prognosis than those with brain metastasis, whose median survival time was 1–8 months [7,8,19,22–25]. In this study, as expected, patients with brain metastasis indicated worst clinical outcome with a median survival month of 6, which is much shorter compared with the lung (9 months) or liver (8.5 months) or bone (11 months) metastasis group. Previous study showed that the median OS for only lung, only liver, only bone, and only brain metastasis was 5, 5, 4, and 3 months, respectively, which was consistent with our results [26].

Additionally, we concluded that patients with grade I–II demonstrated worse OS outcomes than those with grade III–IV. As previous studies showed, brain metastasis was more common in moderately differentiated cervical cancer rather than in well, poorly, and undifferentiated tumors [26]. This might partially explain our results. Further analysis demonstrated that patients synchronously diagnosed with bone and brain metastasis presented with worse clinical outcomes. We supposed that the reason behind this phenomenon was that cervical cancer patients with liver metastasis and with lung metastasis was available for much more treatment choice including surgical resection, chemotherapy, or radiotherapy than with bone metastasis [10,27]. The main treatment modality for patients with bone metastasis was either radiotherapy or chemotherapy. And indications for surgery in these patients were difficult to determine [28]. We retrieved literature and found that only two patients with bone metastasis were treated surgically and complete resection was not possible [28]. In addition to the above-mentioned survival factors, the prognosis of cervical cancer with brain metastasis was also reported to be affected by many factors such as age, pathological type, control of primary cervical lesions, number of brain metastasis and tumor size, extracranial metastasis, time interval from diagnosis of cervical cancer to the diagnosis of brain metastasis, extracranial metastasis, and therapeutic factors [7]. For example, being younger than 50 years of age, in good physical condition, with single brain metastases, and without extracranial metastases was associated with a good prognosis [19]. And presenting extracerebral metastasis, diameter of the largest brain metastasis <2 cm, time between SCC diagnosis and brain metastasis <1 year indicated worse prognosis [8].

As for treatment, we found that chemoradiotherapy could prolong the survival of stage IVB cervical cancer patients with brain metastasis. This result was consistent with previous studies. Multimodal therapy can prolong OS [13,29], consisting of surgery, SRS, WBRT, and chemotherapy. In our research, we found that chemoradiotherapy tended to prolong the survival of stage IVB cervical cancer patients with brain metastasis. Some literature has also found that bevacizumab, when used in conjunction with standard chemotherapy, can increase clinical benefits for cancer patients (such as OS, progression-free survival, and response rates). This suggests that targeted therapies such as anti-angiogenic agents may be considered for cervical cancer patients with brain metastasis to improve OS [30]. However, from literature review, the best option for single brain metastasis from cervical cancer is neurosurgical resection followed by radiation therapy. WBRT is considered the primary palliation treatment for patients with multiple or inoperable brain metastasis [16]. Additionally, it has been indicated that for inoperable ovarian cancer patients, neoadjuvant chemotherapy is a safe and effective alternative. Therefore, for inoperable cervical cancer patients with brain metastasis, neoadjuvant chemotherapy might be employed with the intention of achieving tumor shrinkage before surgical resection; however, this requires further research for validation [31]. Overall, surgical resection combined with radiotherapy can provide the most satisfactory treatment effect for patients with single brain metastasis without systemic disease. While in terms of multiple brain metastases, the use of palliative brain radiotherapy is an optimal choice. Chemotherapy is suitable for multiple brain metastases accompanied with other organ metastasis and systemic disease [13,32,33]. Since information about surgery is not available in the SEER database, further studies are expected to investigate the effect of surgical resection on survival outcomes in patients with brain metastasis.

We must admit that there are some limitations to this study. As this study is a retrospective study, it is inevitable that there will be some bias. In addition, the cases included in this study were strictly screened, and a large number of patients were excluded from the analysis, resulting in missing or incomplete information and further information bias. We do not have access to detailed treatment information of cervical cancer patients, including the type of surgery, radiation dose, chemotherapy drugs, detailed information, for example the number of brain metastasis and so on, which is a major limitation of this study. Due to the limited number of patients, especially those with brain metastases, larger sample sizes and prospective further studies are needed for more comprehensive and comparative analyses. For all this, the study analyzed clinical characteristics of cervical cancer patients with brain metastasis and assessed prognostic factors influencing OS in patients which have certain guiding significance for clinical practice.

In conclusion, the occurrence of brain metastasis in cervical cancer patients indicated poor prognosis. We need more research studies to verify our conclusion and provide more appropriate treatment plans, so as to prolong the survival time and improve the quality of life of such patients.
